# (*E*)-2-(4-Chloro­benzyl­idene)indan-1-one

**DOI:** 10.1107/S1600536811027589

**Published:** 2011-07-16

**Authors:** Mohamed Ashraf Ali, Tan Soo Choon, Lim Yee Lan, Mohd Mustaqim Rosli, Hoong-Kun Fun

**Affiliations:** a Institute for Research in Molecular Medicine, Universiti Sains Malaysia, 11800 USM, Penang, Malaysia; b School of Chemical Sciences, Universiti Sains Malaysia, 11800 USM, Penang, Malaysia; c X-ray Crystallography Unit, School of Physics, Universiti Sains Malaysia, 11800 USM, Penang, Malaysia

## Abstract

In the title compound, C_16_H_11_ClO, the dihedral angle between the almost planar dihydro­indene ring system (r.m.s. deviation = 0.009 Å) and the chloro­benzene ring is 3.51 (14)°. In the crystal, mol­ecules are connected by C—H⋯O and weak C—H⋯Cl inter­actions, forming infinite layers parallel to (101).

## Related literature

For biological background to dihydro­indene derivatives, see: Akritopoulou-Zanze *et al.* (2007 ▶); Muhsin *et al.* (2006 ▶). For the stability of the temperature controller used in the data collection, see: Cosier & Glazer (1986 ▶).
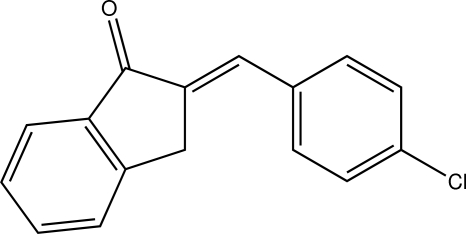


## Experimental

### 

#### Crystal data

C_16_H_11_ClO
*M* *_r_* = 254.70
Triclinic, 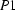

*a* = 3.8649 (2) Å
*b* = 6.5233 (3) Å
*c* = 12.1703 (6) Å
α = 91.374 (4)°
β = 95.914 (4)°
γ = 103.483 (4)°
*V* = 296.43 (3) Å^3^
*Z* = 1
Mo *K*α radiation
μ = 0.30 mm^−1^
*T* = 100 K
0.43 × 0.28 × 0.04 mm

#### Data collection

Bruker SMART APEXII CCD diffractometer
Absorption correction: multi-scan (*SADABS*; Bruker, 2009 ▶) *T*_min_ = 0.882, *T*_max_ = 0.988
3942 measured reflections
2159 independent reflections
2072 reflections with *I* > 2σ(*I*)
*R*_int_ = 0.039

#### Refinement

*R*[*F* ^2^ > 2σ(*F* ^2^)] = 0.046
*wR*(*F* ^2^) = 0.124
*S* = 1.06
2159 reflections
163 parameters
3 restraints
H-atom parameters constrained
Δρ_max_ = 0.63 e Å^−3^
Δρ_min_ = −0.48 e Å^−3^
Absolute structure: Flack (1983 ▶), 870 Friedel pairs
Flack parameter: 0.05 (8)

### 

Data collection: *APEX2* (Bruker, 2009 ▶); cell refinement: *SAINT* (Bruker, 2009 ▶); data reduction: *SAINT*; program(s) used to solve structure: *SHELXTL* (Sheldrick, 2008 ▶); program(s) used to refine structure: *SHELXTL*; molecular graphics: *SHELXTL*; software used to prepare material for publication: *SHELXTL* and *PLATON* (Spek, 2009 ▶).

## Supplementary Material

Crystal structure: contains datablock(s) global, I. DOI: 10.1107/S1600536811027589/hb5928sup1.cif

Structure factors: contains datablock(s) I. DOI: 10.1107/S1600536811027589/hb5928Isup2.hkl

Supplementary material file. DOI: 10.1107/S1600536811027589/hb5928Isup3.cml

Additional supplementary materials:  crystallographic information; 3D view; checkCIF report

## Figures and Tables

**Table 1:** Hydrogen-bond geometry (Å, °)

*D*—H⋯*A*	*D*—H	H⋯*A*	*D*⋯*A*	*D*—H⋯*A*
C1—H1*A*⋯O1^i^	0.99	2.49	3.436 (4)	159
C5—H5*A*⋯Cl1^ii^	0.95	2.80	3.591 (4)	141

Symmetry codes: (i) 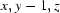; (ii) 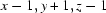.

**Fig. 1.**
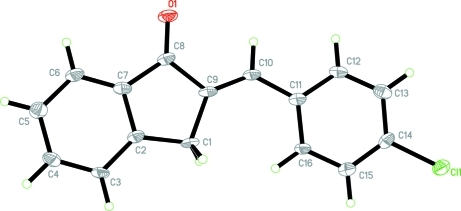
The molecular structure, showing 50% probability displacement ellipsoids. Hydrogen atoms are shown as spheres of arbitrary radius.

**Fig. 2.**
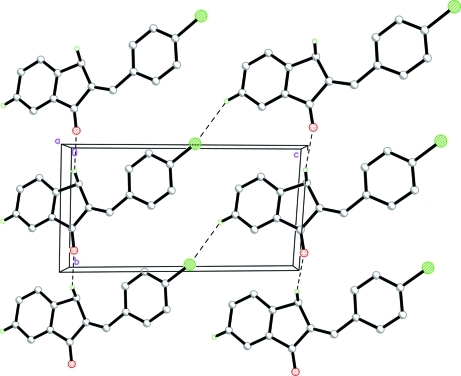
The packing of (I) viewed along the a axis. Dashed lines indicate hydrogen bonds. H atoms not involved in the hydrogen bond interactions have been omitted for clarity.

## supplementary crystallographic information

### Comment

Substituted dihydroindene derivatives have been used as multitargeted kinase inhibitors: Initial efforts focused on the development of selective KDR inhibitors, while later strategies involved the improvement of potency toward multiple kinase targets (Akritopoulou-Zanze *et al.* 2007). Thus,  several dihydroindene derivatives were identified as potent KDR, Flt1, Flt3, and c-Kit inhibitors. Initial strategies involved single target therapies and resulted in the FDA approval of Avastin (a humanized monoclonal antibody targeting VEGF, the growth factor that stimulates VEGFRs) for the treatment of metastatic colorectal cancer (Muhsin *et al.* 2006). As part of our studies in this area, we now report the synthesis and structure of the title compound, (I).

All parameters in (I) within normal ranges. The dihydroindene ring is almost planar with the maximum deviation of -0.015 (4)Å for atom C7. It make a dihedral angle of 3.51 (14)° with the adjacent benzene ring (Fig. 1). In the crystal, the molecules are interconnected by C—H···O and C—H···Cl interactions (Table 1) to form infinite layers (Fig. 2) parallel to the (101)-plane.

### Experimental

A mixture of  2,3-dihydro-IH-indene-1-one (0.001 mmol) and 4-chlorbenzaldehyde (0.001 mmol) were dissolved in methanol (10 mL) and  30% sodium hydroxide solution (5ml) was added. The solution was stirred for 5 hour. After completion of the reaction as evident from TLC, the mixture was poured into crushed ice then neutralized with Con HCl.  The precipitated solid was filtered, washed with water and recrystallised from ethanol to reveal the title compound as light yellow plates of (I).

### Refinement

All H-atoms were positioned geometrically and refined using a riding model, with C-H = 0.95 and 0.99Å, and with U_iso_ = 1.2U_eq_(C).

### Crystal data

**Table:**

C_16_H_11_ClO	*Z* = 1
*M**_r_* = 254.70	*F*(000) = 132
Triclinic, *P*1	*D*_x_ = 1.427 Mg m^−^^3^
Hall symbol: P 1	Mo *K*α radiation, λ = 0.71073 Å
*a* = 3.8649 (2) Å	Cell parameters from 2756 reflections
*b* = 6.5233 (3) Å	θ = 3.2–32.1°
*c* = 12.1703 (6) Å	µ = 0.30 mm^−^^1^
α = 91.374 (4)°	*T* = 100 K
β = 95.914 (4)°	Plate, light-yellow
γ = 103.483 (4)°	0.43 × 0.28 × 0.04 mm
*V* = 296.43 (3) Å^3^	

### Data collection

**Table:**

Bruker SMART APEXII CCD diffractometer	2159 independent reflections
Radiation source: fine-focus sealed tube	2072 reflections with *I* > 2σ(*I*)
graphite	*R*_int_ = 0.039
φ and ω scans	θ_max_ = 27.0°, θ_min_ = 1.7°
Absorption correction: multi-scan (*SADABS*; Bruker, 2009)	*h* = −4→4
*T*_min_ = 0.882, *T*_max_ = 0.988	*k* = −8→8
3942 measured reflections	*l* = −15→15

### Refinement

**Table:**

Refinement on *F*^2^	Secondary atom site location: difference Fourier map
Least-squares matrix: full	Hydrogen site location: inferred from neighbouring sites
*R*[*F*^2^ > 2σ(*F*^2^)] = 0.046	H-atom parameters constrained
*wR*(*F*^2^) = 0.124	*w* = 1/[σ^2^(*F*_o_^2^) + (0.0685*P*)^2^ + 0.1711*P*] where *P* = (*F*_o_^2^ + 2*F*_c_^2^)/3
*S* = 1.06	(Δ/σ)_max_ < 0.001
2159 reflections	Δρ_max_ = 0.63 e Å^−^^3^
163 parameters	Δρ_min_ = −0.48 e Å^−^^3^
3 restraints	Absolute structure: Flack (1983), 870 Friedel pairs
Primary atom site location: structure-invariant direct methods	Flack parameter: 0.05 (8)

### Special details

**Table:**

Experimental. The crystal was placed in the cold stream of an Oxford Cryosystems Cobra open-flow nitrogen cryostat (Cosier & Glazer, 1986) operating at 100.0 (1) K.
Geometry. All esds (except the esd in the dihedral angle between two l.s. planes) are estimated using the full covariance matrix. The cell esds are taken into account individually in the estimation of esds in distances, angles and torsion angles; correlations between esds in cell parameters are only used when they are defined by crystal symmetry. An approximate (isotropic) treatment of cell esds is used for estimating esds involving l.s. planes.
Refinement. Refinement of F^2^ against ALL reflections. The weighted R-factor wR and goodness of fit S are based on F^2^, conventional R-factors R are based on F, with F set to zero for negative F^2^. The threshold expression of F^2^ > 2sigma(F^2^) is used only for calculating R-factors(gt) etc. and is not relevant to the choice of reflections for refinement. R-factors based on F^2^ are statistically about twice as large as those based on F, and R- factors based on ALL data will be even larger.

### Fractional atomic coordinates and isotropic or equivalent isotropic displacement parameters (Å^2^)

**Table:**

	*x*	*y*	*z*	*U*_iso_*/*U*_eq_	
Cl1	1.33869 (16)	−0.02880 (10)	0.53352 (7)	0.0254 (2)	
O1	0.9589 (7)	0.8574 (3)	0.0177 (2)	0.0249 (5)	
C1	0.5969 (8)	0.2910 (5)	0.0183 (3)	0.0179 (7)	
H1A	0.7420	0.1882	0.0046	0.022*	
H1B	0.4062	0.2263	0.0639	0.022*	
C2	0.4406 (9)	0.3621 (5)	−0.0892 (3)	0.0184 (6)	
C3	0.2093 (9)	0.2374 (5)	−0.1742 (3)	0.0194 (7)	
H3A	0.1294	0.0893	−0.1683	0.023*	
C4	0.0999 (9)	0.3344 (5)	−0.2667 (3)	0.0226 (7)	
H4A	−0.0572	0.2512	−0.3248	0.027*	
C5	0.2154 (10)	0.5534 (6)	−0.2770 (3)	0.0239 (7)	
H5A	0.1358	0.6175	−0.3410	0.029*	
C6	0.4483 (9)	0.6754 (5)	−0.1921 (3)	0.0221 (7)	
H6A	0.5322	0.8231	−0.1983	0.026*	
C7	0.5554 (9)	0.5799 (5)	−0.0994 (3)	0.0196 (7)	
C8	0.8022 (8)	0.6717 (5)	−0.0003 (3)	0.0186 (7)	
C9	0.8293 (9)	0.4952 (5)	0.0738 (3)	0.0188 (7)	
C10	1.0366 (9)	0.5332 (5)	0.1708 (3)	0.0195 (7)	
H10A	1.1649	0.6758	0.1856	0.023*	
C11	1.0966 (8)	0.3896 (5)	0.2575 (3)	0.0177 (7)	
C12	1.3093 (9)	0.4750 (5)	0.3561 (3)	0.0205 (7)	
H12A	1.4065	0.6231	0.3639	0.025*	
C13	1.3816 (9)	0.3519 (6)	0.4415 (3)	0.0225 (7)	
H13A	1.5242	0.4136	0.5076	0.027*	
C14	1.2417 (9)	0.1354 (5)	0.4291 (3)	0.0202 (7)	
C15	1.0307 (9)	0.0433 (5)	0.3330 (3)	0.0210 (7)	
H15A	0.9361	−0.1051	0.3259	0.025*	
C16	0.9595 (9)	0.1698 (5)	0.2477 (3)	0.0178 (7)	
H16A	0.8164	0.1071	0.1818	0.021*	

### Atomic displacement parameters (Å^2^)

**Table:**

	*U*^11^	*U*^22^	*U*^33^	*U*^12^	*U*^13^	*U*^23^
Cl1	0.0283 (4)	0.0188 (4)	0.0279 (4)	0.0036 (3)	0.0010 (3)	0.0050 (3)
O1	0.0255 (13)	0.0120 (11)	0.0341 (13)	−0.0017 (10)	0.0027 (10)	0.0018 (9)
C1	0.0187 (16)	0.0079 (13)	0.0265 (16)	−0.0002 (12)	0.0066 (13)	0.0009 (11)
C2	0.0165 (16)	0.0129 (14)	0.0253 (15)	0.0011 (13)	0.0048 (12)	0.0038 (12)
C3	0.0150 (15)	0.0120 (15)	0.0273 (17)	−0.0045 (13)	0.0025 (13)	−0.0001 (12)
C4	0.0166 (16)	0.0209 (16)	0.0263 (16)	−0.0032 (14)	0.0017 (13)	−0.0013 (13)
C5	0.0201 (17)	0.0222 (18)	0.0279 (17)	0.0004 (14)	0.0046 (13)	0.0051 (13)
C6	0.0204 (17)	0.0136 (15)	0.0311 (18)	0.0006 (13)	0.0051 (14)	0.0042 (12)
C7	0.0161 (16)	0.0139 (15)	0.0272 (17)	−0.0006 (12)	0.0052 (13)	−0.0006 (12)
C8	0.0148 (15)	0.0118 (14)	0.0297 (17)	0.0024 (12)	0.0053 (13)	0.0037 (12)
C9	0.0163 (15)	0.0092 (15)	0.0293 (17)	−0.0019 (12)	0.0064 (13)	0.0006 (12)
C10	0.0180 (16)	0.0102 (14)	0.0276 (16)	−0.0023 (12)	0.0031 (13)	0.0005 (12)
C11	0.0146 (16)	0.0121 (15)	0.0262 (17)	0.0021 (12)	0.0040 (13)	0.0006 (12)
C12	0.0181 (16)	0.0131 (15)	0.0283 (17)	0.0003 (13)	0.0015 (13)	−0.0033 (12)
C13	0.0171 (17)	0.0207 (17)	0.0273 (18)	0.0001 (14)	0.0025 (14)	−0.0039 (14)
C14	0.0159 (17)	0.0200 (17)	0.0251 (17)	0.0037 (13)	0.0039 (13)	0.0088 (13)
C15	0.0209 (18)	0.0147 (16)	0.0270 (18)	0.0031 (14)	0.0030 (14)	0.0017 (13)
C16	0.0165 (16)	0.0111 (15)	0.0232 (17)	−0.0012 (13)	0.0014 (13)	0.0000 (12)

### Geometric parameters (Å, °)

**Table:**

Cl1—C14	1.749 (3)	C7—C8	1.478 (5)
O1—C8	1.225 (4)	C8—C9	1.495 (4)
C1—C2	1.513 (5)	C9—C10	1.340 (5)
C1—C9	1.520 (4)	C10—C11	1.464 (5)
C1—H1A	0.9900	C10—H10A	0.9500
C1—H1B	0.9900	C11—C12	1.405 (4)
C2—C7	1.399 (4)	C11—C16	1.406 (4)
C2—C3	1.400 (4)	C12—C13	1.375 (5)
C3—C4	1.382 (5)	C12—H12A	0.9500
C3—H3A	0.9500	C13—C14	1.388 (5)
C4—C5	1.407 (5)	C13—H13A	0.9500
C4—H4A	0.9500	C14—C15	1.391 (5)
C5—C6	1.395 (5)	C15—C16	1.386 (5)
C5—H5A	0.9500	C15—H15A	0.9500
C6—C7	1.375 (5)	C16—H16A	0.9500
C6—H6A	0.9500		
			
C2—C1—C9	103.1 (3)	C7—C8—C9	107.3 (3)
C2—C1—H1A	111.2	C10—C9—C8	120.3 (3)
C9—C1—H1A	111.2	C10—C9—C1	131.1 (3)
C2—C1—H1B	111.2	C8—C9—C1	108.5 (3)
C9—C1—H1B	111.2	C9—C10—C11	130.2 (3)
H1A—C1—H1B	109.1	C9—C10—H10A	114.9
C7—C2—C3	119.8 (3)	C11—C10—H10A	114.9
C7—C2—C1	112.3 (3)	C12—C11—C16	117.6 (3)
C3—C2—C1	127.8 (3)	C12—C11—C10	118.4 (3)
C4—C3—C2	118.6 (3)	C16—C11—C10	124.0 (3)
C4—C3—H3A	120.7	C13—C12—C11	122.4 (3)
C2—C3—H3A	120.7	C13—C12—H12A	118.8
C3—C4—C5	121.6 (3)	C11—C12—H12A	118.8
C3—C4—H4A	119.2	C12—C13—C14	118.5 (3)
C5—C4—H4A	119.2	C12—C13—H13A	120.8
C6—C5—C4	119.2 (3)	C14—C13—H13A	120.8
C6—C5—H5A	120.4	C13—C14—C15	121.3 (3)
C4—C5—H5A	120.4	C13—C14—Cl1	120.2 (2)
C7—C6—C5	119.4 (3)	C15—C14—Cl1	118.5 (3)
C7—C6—H6A	120.3	C16—C15—C14	119.5 (3)
C5—C6—H6A	120.3	C16—C15—H15A	120.3
C6—C7—C2	121.3 (3)	C14—C15—H15A	120.3
C6—C7—C8	129.9 (3)	C15—C16—C11	120.7 (3)
C2—C7—C8	108.7 (3)	C15—C16—H16A	119.6
O1—C8—C7	126.5 (3)	C11—C16—H16A	119.6
O1—C8—C9	126.1 (3)		
			
C9—C1—C2—C7	−0.5 (4)	O1—C8—C9—C1	−178.8 (3)
C9—C1—C2—C3	178.9 (3)	C7—C8—C9—C1	0.6 (3)
C7—C2—C3—C4	0.0 (5)	C2—C1—C9—C10	−179.6 (3)
C1—C2—C3—C4	−179.4 (3)	C2—C1—C9—C8	−0.1 (3)
C2—C3—C4—C5	0.0 (5)	C8—C9—C10—C11	177.9 (3)
C3—C4—C5—C6	0.5 (5)	C1—C9—C10—C11	−2.6 (6)
C4—C5—C6—C7	−1.1 (5)	C9—C10—C11—C12	−175.1 (3)
C5—C6—C7—C2	1.2 (5)	C9—C10—C11—C16	6.1 (6)
C5—C6—C7—C8	178.7 (3)	C16—C11—C12—C13	−0.7 (5)
C3—C2—C7—C6	−0.6 (5)	C10—C11—C12—C13	−179.6 (3)
C1—C2—C7—C6	178.9 (3)	C11—C12—C13—C14	0.7 (5)
C3—C2—C7—C8	−178.6 (3)	C12—C13—C14—C15	−0.4 (5)
C1—C2—C7—C8	0.9 (4)	C12—C13—C14—Cl1	177.8 (3)
C6—C7—C8—O1	0.7 (5)	C13—C14—C15—C16	0.3 (5)
C2—C7—C8—O1	178.5 (3)	Cl1—C14—C15—C16	−178.0 (3)
C6—C7—C8—C9	−178.6 (3)	C14—C15—C16—C11	−0.3 (5)
C2—C7—C8—C9	−0.9 (4)	C12—C11—C16—C15	0.5 (5)
O1—C8—C9—C10	0.8 (5)	C10—C11—C16—C15	179.3 (3)
C7—C8—C9—C10	−179.8 (3)		

### Hydrogen-bond geometry (Å, °)

**Table:**

*D*—H···*A*	*D*—H	H···*A*	*D*···*A*	*D*—H···*A*
C1—H1A···O1^i^	0.99	2.49	3.436 (4)	159
C5—H5A···Cl1^ii^	0.95	2.80	3.591 (4)	141

Symmetry codes: (i) *x*, *y*−1, *z*; (ii) *x*−1, *y*+1, *z*−1.
